# The Relationship between Visceral Fat Accumulation and Risk of Cardiometabolic Multimorbidity: The Roles of Accelerated Biological Aging

**DOI:** 10.3390/nu17081397

**Published:** 2025-04-21

**Authors:** Tianyu Zhu, Yixing Tian, Jinqi Wang, Zhiyuan Wu, Wenhan Xie, Haotian Liu, Xia Li, Lixin Tao, Xiuhua Guo

**Affiliations:** 1 Beijing Key Laboratory of Environment and Aging, School of Public Health, Capital Medical University, No. 10 Xitoutiao, Youanmen Street, Beijing 100069, China; 2 Department of Nutrition, Harvard T.H. Chan School of Public Health, 655 Huntington Avenue, Boston, MA 02115, USA; 3 Department of Mathematics and Statistics, La Trobe University, Melbourne, VIC 3086, Australia; 4 Centre for Precision Health, School of Medical and Health Sciences, Edith Cowan University, 270 Joondalup Drive, Joondalup, WA 6027, Australia; 5 Department of Epidemiology and Health Statistics, School of the Public Health, Capital Medical University, No. 10 Xitoutiao, Youanmen Street, Beijing 100069, China

**Keywords:** body roundness index, visceral obesity, cardiometabolic multimorbidity, accelerated biological aging

## Abstract

Objectives: To investigate the association between visceral fat accumulation and the risk of cardiometabolic multimorbidity (CMM) and the potential roles of accelerated biological aging in this relationship. Methods: Using data from the UK Biobank, a nationwide cohort study was conducted using the available baseline body roundness index (BRI) measurement. Biological aging was assessed using the Klemera–Doubal method for biological age and the phenotypic age algorithms. The association between the BRI and CMM was estimated using the Cox proportional hazards regression model, while the roles of biological aging were examined through interaction and mediation analyses. Results: During a median follow-up of 14.52 years, 6156 cases of CMM were identified. A significant association was observed between the BRI and CMM. The hazard ratio (HR) for CMM was 3.72 (95% confidence interval [CI]: 3.35–4.13) for individuals in the highest quartile compared with those in the lowest quartile of the BRI. More importantly, the BRI (AUC, 0.701; 95% CI, 0.694–0.707) demonstrated superior predictive performance relative to body mass index (AUC, 0.657; 95% CI, 0.650–0.664). Furthermore, the BRI exhibited additive interactions with accelerated biological aging on the risk of CMM, and accelerated biological aging partially mediated the association between the BRI and CMM. Conclusions: These findings provide evidence for the application of the BRI as a novel and readily accessible screening tool associated with CMM, suggesting that the effective management of visceral fat and biological aging deceleration may hold promise for reducing CMM risk.

## 1. Introduction

Cardiometabolic multimorbidity (CMM), as one of the most prevalent and representative multimorbidity forms, is characterized by the coexistence of at least two cardiometabolic diseases (CMD), typically including coronary heart disease (CHD), type 2 diabetes (T2DM), and stroke [1,2]. Compared to single CMD, patients with CMM have been found to face a multiplicative risk of mortality and a marked reduction in life expectancy [1]. The existing evidence also supports that CMM is associated with cognitive decline [3] and depression in later life [4]. The prevalence of CMM among the population aged 60 years and older has been reported to be 2.5-fold higher than that among those aged 40 years and older [2], with approximately 30% of older adults affected [5]. Even more concerning is that the number of elders is projected to reach 1.5 billion by 2050 [6], and these issues will be further exacerbated by the aging population. Therefore, from both individual and public health perspectives, identifying modifiable risk factors of CMM is now an urgent priority.

Obesity is widely recognized as a significant risk factor for CMM. Generally, the relationship between obesity and CMM is assessed using body mass index (BMI) as an indicator [7,8]. With extensive research into body composition, body fat content has garnered increased attention. A growing consensus holds that the accumulation of visceral adipose tissue is more deleterious to health than the expansion of subcutaneous adipose tissue [9]. However, BMI is limited by its inability to differentiate between fat mass and muscle mass [10]. The body roundness index (BRI), introduced by Thomas et al., is a novel anthropometric measure that integrates height and waist circumference (WC) to characterize body shape. Compared to the traditional BMI, it provides a more accurate reflection of visceral fat distribution [11]. In addition, previous studies have shown that the BRI outperforms BMI in predicting multiple clinical endpoints, including diabetes, cardiovascular disease (CVD), and mortality [12,13,14]. However, the association between the BRI and the risk of CMM has yet to be explored. It remains uncertain whether the BRI demonstrates superior predictive capacity over BMI in predicting CMM. Furthermore, the underlying mechanisms through which the BRI influences CMM remain largely unrevealed.

Aging, a complex biological process, is the gradual deterioration of the integrity, reserve function, and recovery capacity of cells, tissues, and organs [15]. Compared to chronological age, biological age provides a more accurate reflection of an individual’s true aging status and risk of age-related diseases [16]. The Klemera–Doubal method for biological age (KDM-BA), introduced by Klemera and Doubal [17], and phenotypic age (PhenoAge) proposed by Levine et al. [18] both utilize clinical measurements and blood biomarkers, offering a comprehensive evaluation of biological age. The phenomenon in which the biological age of an individual advances more rapidly than their chronological age is referred to as accelerated aging [19]. Essentially, biological aging, linked to reduced metabolic rates, vascular stiffness, low-level chronic inflammation, oxidative stress, and the interplay of comorbidities, may play a critical role in the pathology of CMM [20,21]. On the other hand, the accumulation of predominantly pro-inflammatory immune cells and alterations in the immune profile of adipose tissue in obese individuals potentially act as potent drivers of accelerated aging [22,23]. Despite these insights, no research has simultaneously examined the relationship between BRI, biological aging, and CMM.

Therefore, this study aims to assess the association between the BRI and CMM risk, as well as the predictive performance of the BRI for CMM. Furthermore, the roles of biological aging in BRI-associated CMM will be explored.

## 2. Materials and Methods

### 2.1. Study Population

The UK Biobank, a large-scale prospective cohort study, recruited more than 500,000 participants across 22 assessment centers between 2006 and 2010, collecting a comprehensive range of data on lifestyle factors, anthropometric measurements, biological samples, and health status [24]. The study design and implementation have been described in detail in previous publications [24]. All participants provided informed consent and authorized the linkage of national datasets, including primary care, hospital, cancer registry, and death records, to track health-related outcomes. Ethical approval of UK Biobank was granted by the North West Multi-Centre Research Ethics Committee (reference: 21/NW/0157). This research was conducted under application number “88589”.

**Figure 1:**
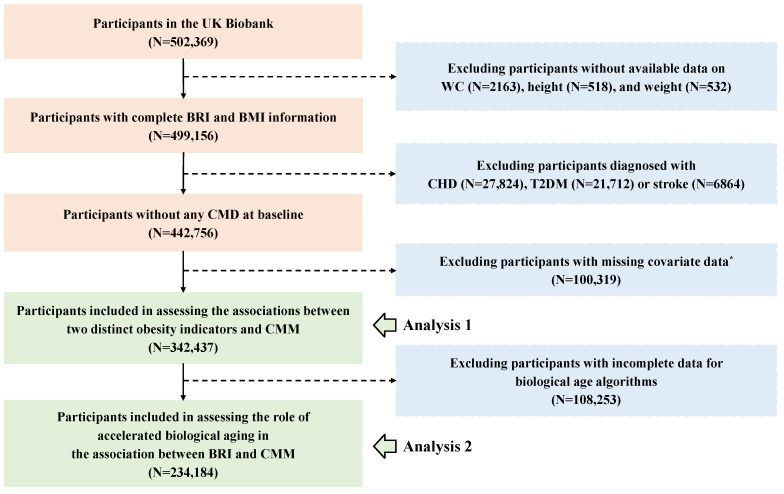
The flowchart for the participants’ inclusion and exclusion. Abbreviations: WC—waist circumference; BRI—body roundness index; BMI—body mass index; CHD—coronary heart disease; T2DM—type 2 diabetes; CMD—cardiometabolic disease; CMM—cardiometabolic multimorbidity. *, Due to a high proportion (22.66%) missing of the International Physical Activity Questionnaire (IPAQ), participants with incomplete physical activity information were excluded. The remaining covariates were imputed using random forest method before analysis.

In our study, participants who met the following criteria were excluded: first, those without available data on WC (*n* = 2163), height (*n* = 518), and weight (*n* = 532); and second, participants diagnosed with any CMD before recruitment (*n* = 56,400). Additionally, for the physical activity covariate, which had a high proportion of missing data (22.66%), we chose not to perform imputation; so 100,319 participants were excluded. Hence, the association between the BRI and CMM was analyzed in 342,437 participants. Furthermore, 108,253 participants who lacked clinical biomarkers data for the calculation of biological age were excluded, leaving 234,184 participants in the analysis to assess the role of accelerated biological aging in the association between the BRI and CMM. The detailed flowchart is presented in Figure 1.

### 2.2. BRI Definition

The BRI was calculated using height (centimeter, cm) and WC (cm), based on the formula previously published by Thomas et al. [11]. The formula is presented below:

$$\mathrm{BRI}=364.2-365.5*\sqrt{\frac{{\left(\frac{\mathrm{WC}}{2\pi }\right)}^{2}}{{\left(0.5*\mathrm{height}\right)}^{2}}}$$

### 2.3. Ascertainment of Outcome

The outcome of the present study was CMM, defined as the coexistence of at least two of the following CMDs: CHD, T2DM, and stroke, consistent with several previous studies [6,7,25]. The onset date of CMM was defined as the date when the second incident CMD was diagnosed. The occurrence of relevant events was ascertained through primary healthcare, hospitalization, and other records, and subsequently confirmed using the codes from the 9th and 10th editions of the International Classification of Diseases (ICD-9 and ICD-10) (Table S1).

### 2.4. Definitions of Covariates

To account for potential confounding factors, several key covariates were incorporated. Demographic factors included age (<60 years, ≥60 years), sex (female and male), race, socioeconomic status, and education level (high school or below, college degree or above). Socioeconomic status was measured using the Townsend Deprivation Index (TDI), which combines multiple dimensions, such as social class, employment, car availability, and household overcrowding [26]. A higher TDI score suggests a greater degree of deprivation. Using the median TDI score, participants were classified into high and low socioeconomic status. Health behavior factors included smoking status (never, former, or current), moderate alcohol intake (yes and no), and physical activity (low, moderate, or high). Moderate alcohol consumption was defined as ≤14 g per day for women or ≤28 g per day for men [27]. Physical activity was evaluated using the International Physical Activity Questionnaire (IPAQ), and participants were categorized into three groups: low (<600 min/week), moderate (600–3000 min/week), and high (>3000 min/week) [28]. We also considered baseline hypertension (yes and no) and dyslipidemia (yes and no) based on medical treatment, self-report, and hospital records.

### 2.5. Biological Aging Assessment

The KDM-BA and PhenoAge are two validated and widely used clinical–parameter biological age algorithms, which can be implemented using the available data from the UK Biobank [18,29]. In this study, we constructed both metrics simultaneously to offer a more comprehensive assessment of biological aging by leveraging the strengths of each algorithm (Table S2).

The KDM-BA represents the average biological state corresponding to a given chronological age within a reference population [30]. Briefly, it was derived from a series of regressions of nine selected biomarkers (forced expiratory volume in one second, systolic blood pressure, albumin, alkaline phosphatase, blood urea nitrogen, total cholesterol, creatinine, C-reactive protein, and glycated hemoglobin) against chronological age [31]. On the other hand, the PhenoAge is based on the average biological state associated with specific mortality risk levels in a reference population [30]. It was derived from multivariate analysis of mortality hazards, utilizing nine biomarkers (white blood cell count, red blood cell distribution width, lymphocyte ratio, mean cell volume, albumin, alkaline phosphatase, creatinine, C-reactive protein, and glucose), four of which overlap with KDM-BA [31]. Biological age values were calculated using the R package BioAge [30].

To capture the biological aging status of individuals, we calculated KDM-BA acceleration and PhenoAge acceleration, defined as the residuals from regressing KDM-BA or PhenoAge onto chronological age at the time of biomarkers measurement. Biological age acceleration was classified as KDM-BA or PhenoAge acceleration greater than 0, indicating accelerated aging, while values less than or equal to 0 were considered non-accelerated aging [32].

### 2.6. Statistical Analysis

The baseline characteristics of participants with or without CMM were presented as frequency (percentage) for categorical variables and mean (standard deviation, SD) for continuous variables. Restricted cubic spline (RCS) regression models were constructed based on the Akaike Information Criterion (AIC), and exposure–response curves for BRI and BMI in relation to CMM risk were visualized. Random forest imputation was employed to handle missing data for covariates. The distribution of the imputed values for each variable was consistent with that of the original data. Unless otherwise stated, all analyses were conducted using the imputed data. After testing the proportional hazards assumption, Cox proportional hazards regression models were used to estimate the associations between two distinct obesity indicators and the risk of CMM, with hazard ratios (HR) and 95% confidence intervals (CI) calculated. Additionally, by examining receiver operating characteristic (ROC) curves and calculating the area under the curve (AUC), we determined which had superior discriminatory capacity in predicting CMM between visceral fat content indicator (BRI) and traditional obesity indicator (BMI). To identify subgroups in whom the effect of BRI was noteworthy, stratified analyses were conducted by sex (male vs. female), age (<60 years vs. ≥60 years), race (white vs. other), BMI (<30 kg/m^2^ vs. ≥30 kg/m^2^), economic status (high vs. low), education level (university or above vs. high school or below), moderate alcohol intake (yes vs. no), smoking status (current/former vs. never), and physical activity (active vs. inactive).

Linear regression models were used to evaluate the association between BRI and biological aging, while Cox regression models were employed to assess the relationship between biological aging and the risk of CMM. Based on binary BRI (≤ median value, > median value) and biological age acceleration (≤0, >0), categorical variables were generated to illustrate their joint associations with CMM. To quantify the multiplicative interaction on CMM risk, a product term of these two factors was included in the Cox regression model. The relative excess risk due to interaction (RERI) along with 95% CI was calculated to estimate potential additive interaction [33]. Furthermore, following the VanderWeele method [34], the total effect of BRI on CMM was decomposed into two components: the natural indirect effect (i.e., the effect of BRI mediated through biological aging) and the natural direct effect (i.e., the effect of BRI not explained by biological aging) [35]. Mediation analysis used the R CMAverse package [36].

A series of sensitivity analyses were conducted to test the robustness of the association results. First, the competing risk analysis was performed, treating all-cause mortality as a competing event rather than a censored event, to minimize bias from competing risks that could influence the occurrence of the events of interest [37]. Second, participants diagnosed with CMM within two years of enrollment were excluded to reduce the potential impact of reverse causality. Third, participants with a history of cancer were excluded. Fourth, participants with incomplete covariates information were excluded. Additionally, inverse probability weighting was applied to the participants included in the biological aging analysis to minimize potential selection bias [38]. Specifically, logistic regression was used to calculate the probability of inclusion in the analysis sample, and the inverse of probabilities was applied as weights in the Cox regression model to evaluate the relationship between the BRI, biological aging, and CMM. All analyses were conducted using R software (version 4.4.2), and a two-tailed *p*-value < 0.05 was considered statistically significant.

## 3. Results

### 3.1. Baseline Characteristics

A total of 342,437 eligible participants were included, consisting of 156,677 (45.75%) males and 185,760 (54.25%) females. During a median follow-up period of 14.52 years, 6156 cases of incident CMM were identified. Among the 234,184 participants with complete biological age information, the mean (SD) values for KDM-BA and PhenoAge were 51.92 (12.50) and 49.12 (9.16) years. The baseline characteristics of the participants are provided in Table 1. A significant difference was observed between the incident CMM and non-CMM group in terms of age, sex, education level, economic status, smoking, moderate alcohol intake, physical activity, and history of hypertension and dyslipidemia (all *p* < 0.001). We also found that, compared to the non-CMM group, participants in the CMM group had higher KDM-BA, PhenoAge, KDM-BA acceleration, and PhenoAge acceleration.

### 3.2. BRI Serves as an Effective Predictor for CMM

**Figure 2:**
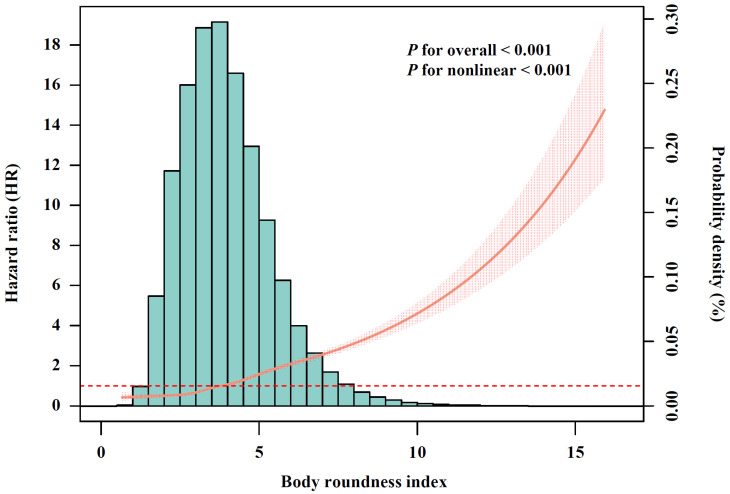
The relationship of body roundness index with cardiometabolic multimorbidity risk after full adjustment (N = 342,437). The solid curve line represents the effect-size estimates for the association, and the light shadow represents the 95% confidence interval. All models were adjusted for age (<60, ≥60 years), sex (female, male), race, education (college degree or above, high school or below), Townsend Deprivation Index (low economic level, high economic level), smoking status (never, previous, or current), moderate alcohol consumption (yes, no), IPAQ (low, moderate, or high), baseline hypertension (yes, no), and baseline dyslipidemia (yes, no).

After conducting multivariable-adjusted RCS analysis (Figure 2), a significant nonlinear association between BRI and incident CMM was observed (*p* for nonlinear < 0.001). Given that no clear cutoff points have been established, BRI was categorized into quartiles, with Q1 representing the lowest and Q4 the highest. Compared with participants in BRI-Q1, those in Q2 (HR, 1.57; 95% CI, 1.40–1.76), Q3 (HR, 2.16; 95% CI, 1.94–2.41) and Q4 (HR, 3.72; 95% CI, 3.35–4.13) exhibited significantly increased risk of CMM (Table 2). The association between BMI and CMM risk is shown in Figure S1 and Table S3. We further calculated the AUC to assess the discriminatory capacities of BRI and BMI in predicting CMM (Figure S2). The results shown that BRI demonstrated superior performance (AUC, 0.701; 95% CI, 0.694–0.707) compared with BMI (AUC, 0.657; 95% CI, 0.650–0.664), with the difference achieving statistically significant (*p* for difference in AUC < 0.001).

### 3.3. Association Between BRI and Biological Aging

Given that biological aging may play a crucial role in the process of CMM onset induced by visceral fat accumulation, we further examined the association between BRI and biological aging. After excluding participants with incomplete data on clinical biomarkers used to calculate biological age, significant associations between BRI and biological age acceleration were identified. The R^2^ value from the linear regression for the correlation between BRI and KDM-BA acceleration was 0.0725, indicating that 7.25% of the variation in KDM-BA acceleration was explained by changes in BRI in the model. For PhenoAge acceleration, BRI explained 3.96% of the variation (Figure S3). In the fully adjusted Model III, participants in Q2, Q3, and Q4 of BRI had significant increases in KDM-BA acceleration of 2.47 years (95% CI, 2.36–2.57), 4.52 years (95% CI, 4.41–4.63), and 7.33 years (95% CI, 7.21–7.44), compared to those in Q1. Regarding PhenoAge acceleration, the increases were 0.20 years (95% CI, 0.15–0.25), 0.57 years (95% CI, 0.52–0.62), and 1.51 years (95% CI, 1.46–1.56), respectively (Table S4).

### 3.4. Association of Biological Aging with Risk of CMM

Table S5 shows the associations of accelerated biological aging with CMM risk. For each 1-year increase in KDM-BA acceleration, the risk of CMM increased by 4% (95% CI, 1.04–1.04), while each 1-year increase in PhenoAge acceleration was associated with a 6% (95% CI, 1.06–1.07) increase in the risk of CMM. The associations remained in models using quartiles of biological age acceleration, with participants in Q2, Q3 and Q4 of KDM-BA or PhenoAge acceleration showing a significantly increased risk of CMM compared to those in Q1.

### 3.5. Role of Accelerated Biological Aging in the Association of BRI with CMM

**Figure 3:**
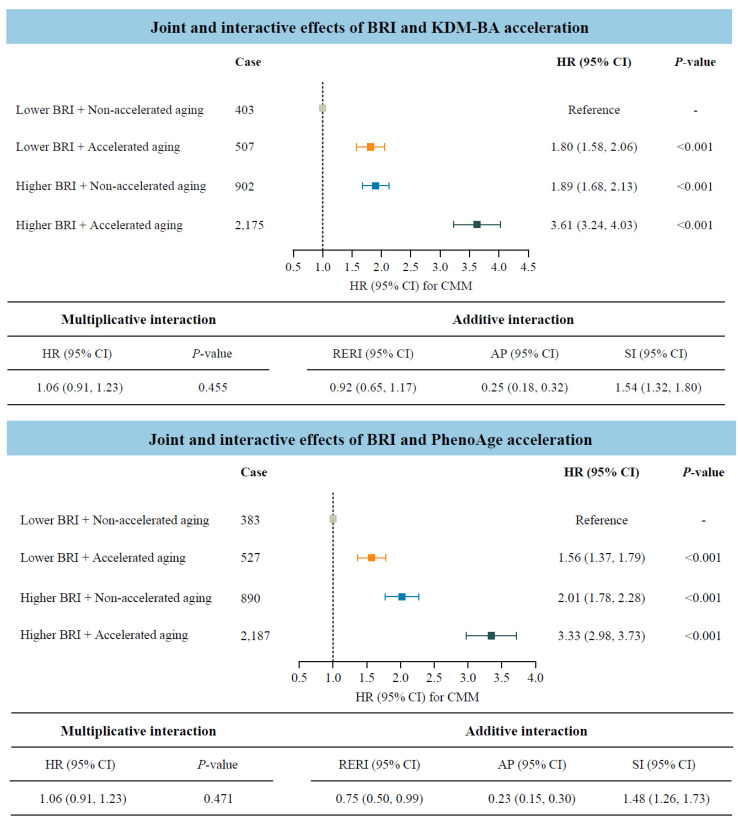
Joint and interactive effects of body roundness index and accelerated biological aging on the risk of cardiometabolic multimorbidity (N = 234,184). Abbreviations: BRI—body roundness index; KDM-BA—Klemera–Doubal method biological age; HR—hazard ratio; CI—confidence interval; CMM—cardiometabolic multimorbidity; RERI—relative excess risk due to interaction; AP—attributable proportion; SI—synergy index; PhenoAge—phenotypic age. All models were adjusted for age (<60, ≥60 years), sex (female, male), race, education (college degree or above, high school or below), Townsend Deprivation Index (low economic level, high economic level), smoking status (never, previous, or current), moderate alcohol intake (yes, no), IPAQ (low, moderate, or high), baseline hypertension (yes, no), and baseline dyslipidemia (yes, no).

Considering that both BRI and accelerated biological aging were potential risk factors for CMM, we further investigated their joint and interactive effects (Figure 3). Participants with accelerated biological aging, measured by KDM-BA or PhenoAge acceleration, and higher BRI indicated significantly higher risks of CMM compared to those with lower BRI and non-accelerated aging. The HRs (95% CIs) were 3.61 (3.24–4.03) and 3.33 (2.98–3.73), respectively. In addition, the statistically significant RERI values indicated positive additive interactions between BRI and biological aging on CMM risk. Specifically, for participants with higher BRI and accelerated biological aging, the corresponding RERI was 0.92 (95% CI: 0.65–1.17) when measured by KDM-BA, and 0.75 (95% CI: 0.50–0.99) when measured by PhenoAge.

**Figure 4:**
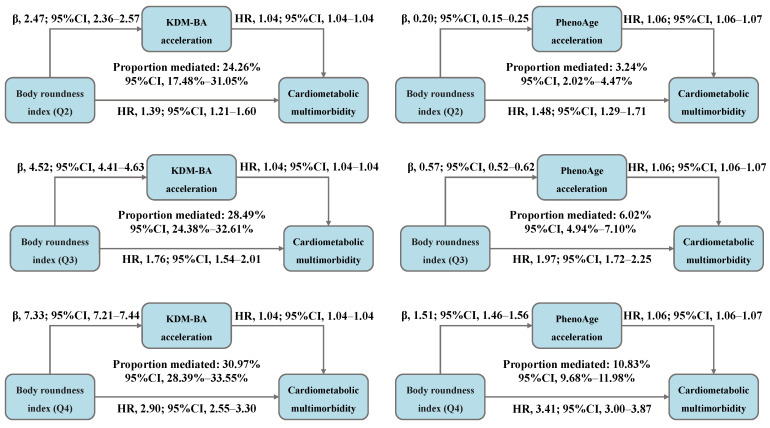
Decomposing the total association between body roundness index and cardiometabolic multimorbidity into direct and indirect associations mediated by accelerated biological aging (N = 234,184). Abbreviations: β—beta coefficient; CI—confidence interval; HR—hazard ratio; Q—quantile; KDM-BA—Klemera–Doubal method biological age; PhenoAge—phenotypic age. All models were adjusted for age (<60, ≥60 years), sex (female, male), race, education (college degree or above, high school or below), Townsend Deprivation Index (low economic level, high economic level), smoking status (never, previous, or current), moderate alcohol intake (yes, no), IPAQ (low, moderate, or high), baseline hypertension (yes, no), and baseline dyslipidemia (yes, no).

The total association between BRI and CMM risk was decomposed into direct and indirect associations mediated by accelerated biological aging, with the results presented in Figure 4 and Table S6. We observed that, compared to BRI-Q1, 24.26%, 28.49%, and 30.97% of the CMM risk in the Q2, Q3, and Q4 was mediated by KDM-BA acceleration, with the HR (95% CI) for indirect association of 1.09 (1.08–1.10), 1.17 (1.16–1.19), and 1.29 (1.27–1.32), respectively. Moreover, 3.24%, 6.02%, and 10.83% of the risk was mediated by PhenoAge acceleration, with the HR (95% CI) for indirect association of 1.01 (1.01–1.01), 1.03 (1.03–1.04), and 1.09 (1.08–1.09), respectively.

### 3.6. Additional and Sensitivity Analyses

Stratified analysis demonstrated that the association between BRI and CMM was consistently pronounced across all subgroups, including participants with BMI < 30 kg/m^2^ and BMI ≥ 30 kg/m^2^ (Table S7).

Sensitivity analyses further confirmed the robustness of these findings, with significant estimates remaining after using a competing risk model accounting for mortality, excluding participants diagnosed with CMM within 2 years of follow-up, excluding participants with a history of cancer, excluding those with missing covariate data, and applying an inverse probability weighted analysis (Tables S8–S10).

## 4. Discussion

In this large-scale, population-based cohort study, two novel findings emerged. Visceral fat accumulation was identified as a strong risk factor for CMM. More importantly, compared with BMI, BRI demonstrated superior predictive performance. Furthermore, individuals with higher BRI and accelerated biological aging exhibited a higher risk of incident CMM, and accelerated biological aging may serve as a partial mediator in the association between BRI and CMM. Overall, these findings provide valuable insights into the complex relationship between the BRI, biological aging, and CMM.

Due to the time-consuming, costly, and impractical nature of conventional methods for visualizing adipose tissue, a simple and effective alternative indicator has been proposed to assess visceral obesity. Theoretically, assuming body shape approximates an ellipse with height as the long axis and WC as the short axis, BRI can be calculated as the eccentricity of the ellipse through human modeling [14]. One thing to add is that BRI can also be useful for people with higher muscle mass, whose BMI might classify them as overweight, while BRI provides a clearer picture of their actual health risks. Therefore, we reasonably hypothesize that BRI provides a more accurate estimation of visceral fat [11].

Several studies have confirmed the associations of BRI with diabetes and CVD incidence across diverse populations. A long-term follow-up study conducted by Liu et al., involving 6990 hypertensive adults without diabetes, found that the BRI was superior to other anthropometric measures in estimating diabetes onset. Furthermore, hypertensive patients with a BRI > 4.62, regardless of their general obesity status, exhibited an elevated diabetes risk [12]. A nationally representative cohort study involving individuals with diabetes or prediabetes in the United States, found that maintaining BRI level at threshold values significantly reduced risks of adverse outcomes, including all-cause and cardiovascular disease-related mortality [13]. Extending these findings, our study provides evidence supporting the significance of BRI in potentially contributing to CMM. It is noteworthy that our study also indicates that BRI outperforms BMI in predicting the incidence of CMM. The poor performance of BMI may be explained by changes in body composition of patients, such as increased total fat mass and decreased muscle mass or bone density, which may result in its classification as normal despite underlying health risks [39,40].

The existing evidence indirectly supports the finding that visceral fat may modulate aging. Interventions aimed at delaying or restricting adipose tissue turnover, redistribution, or dysfunction in experimental animals have been demonstrated to be associated with an extension of maximum healthy lifespan [23]. Also, a secondary analysis of a randomized clinical trial demonstrated that calorie restriction can delay aging [41], partly by blunting the production of pro-inflammatory cytokines from adipose tissue [42], thereby improving adipose tissue metabolic health and function. Additionally, population intervention studies conducted in adults with obesity found telomere lengthening [43] and a reduction in deoxyribonucleic acid methylation age [44]. In our study, established clinical biomarker-based indicators were utilized to measure biological aging, and a positive correlation between BRI and both KDM-BA acceleration and PhenoAge acceleration was consistently observed, providing novel evidence for the association of BRI with biological aging. Epidemiological studies suggest that accelerated biological aging may serve as a useful indicator for identifying high-risk individuals, particularly those predisposed to CMM [45]. Therefore, our study focuses on whether biological aging plays a significant role in the relationship between visceral fat accumulation and CMM risk.

Mechanistically, excessive visceral fat disrupts metabolic homeostasis and glucolipid metabolism while inhibiting insulin signaling and reducing sensitivity, partly due to decreased adiponectin levels and leptin resistance [46,47]. The aberrant metabolic process increases basal lipolysis, leading to the release of free fatty acids, interleukins, and cytokines, which impair inflammatory responses, endothelial function, and coagulation mechanisms, thereby exacerbating vascular and cardiac dysfunction [48]. Higher KDM-BA and PhenoAge acceleration values, which serve as representative biomarkers of abnormality, reflect the unhealthy state stemming from multisystem dysregulation [49]. Specifically, KDM-BA and PhenoAge are composite indices derived from clinical biomarkers that span multiple physiological domains, including inflammation (e.g., C-reactive protein), immunity (e.g., immune cell counts), and organ function (e.g., albumin) [29,50]. Furthermore, obesity aggravates immunosenescence by promoting the activation and differentiation of immune cells within the microvascular environment [51]. Metabolic dysregulation, insulin resistance, and compromised immune function collectively contribute to a pro-aging state that perpetuates a chronic low-grade inflammatory environment and promotes age-related vascular pathology [52,53]. Therefore, biological aging emerges as a plausible mechanistic link between visceral fat accumulation and CMM.

Our findings provide epidemiological support for the biologically plausible hypothesis that biological aging plays a critical role in the pathway linking visceral fat accumulation to CMM. We revealed that the higher risk of CMM occurred with higher BRI and accelerated aging, and that accelerated biological aging partially mediated the relationship between BRI and CMM. The differences in the mediation proportions between the two aging scales may be attributed to the distinct quantification methods. As mentioned earlier, KDM-BA models biological age as the average biological state associated with a particular chronological age, whereas PhenoAge models biological age as the average biological state associated with a specific level of mortality risk in the reference population [30]. Furthermore, the two measures capture distinct aging domains. A genome-wide association study indicated that genes associated with KDM-BA acceleration were enriched in lipid related pathways, while genes related to PhenoAge acceleration showed enrichment for pathways involved in immune system, cell function, and carbohydrate homeostasis [54].

Our findings should be interpreted in the context of some limitations. First, BRI is a dynamic measure, status transitions over time are possible. Future studies focusing on the long-term trajectories of BRI in relation to CMM within well-defined cohorts are imperative, as they will enhance our understanding of the impact and provide more targeted interventions. Second, considering that the UK Biobank cohort is predominantly white, caution should be exercised when generalizing our findings to other regions or racial groups, and further validation is necessary. Third, the relationship between BRI, biological aging, and CMM were analyzed in a subset of the cohort. However, inverse probability weighting was employed to adjust the weights of participants in the biological aging analysis, thereby minimizing potential selection bias and confirming the robustness of the results.

## 5. Conclusions

Our findings provide evidence for the application of BRI as a non-invasive and easily accessible screening tool to estimate the risk of CMM and identify high-risk individuals. Moreover, this study offers an in-depth insight into the role of accelerated biological aging in the relationship between BRI and CMM. In summary, our study aims to raise public and clinical awareness of the BRI as an emerging concept and advocate for effective obesity management with an emphasis on targeting visceral fat beyond simply maintaining BMI within the normal range, while also decelerating biological aging to mitigate the risk of CMM.

## Figures and Tables

**Table 1:** Baseline demographic and health characteristics of study participants stratified by the occurrence of cardiometabolic multimorbidity (N = 342,437).

Characteristics	Overall (N = 342,437)	Incident CMM (N = 6156)	No Incident CMM (N = 336,281)	*p*-Value
Age, *n* (%)				
<60 years	208,483 (60.88)	2088 (33.92)	206,395 (61.38)	<0.001
≥60 years	133,954 (39.12)	4068 (66.08)	129,886 (38.62)	
Sex, *n* (%)				
Female	185,760 (54.25)	2203 (35.79)	183,557 (54.58)	<0.001
Male	156,677 (45.75)	3953 (64.21)	152,724 (45.42)	
Race, *n* (%)				0.151
White	312,134 (91.15)	5579 (90.63)	306,555 (91.16)	
Others	30,303 (8.85)	577 (9.37)	29,726 (8.84)	
Socioeconomic status ^Δ^, *n* (%)				
Low economic level	171,737 (50.15)	3473 (56.42)	168,264 (50.04)	<0.001
High economic level	170,700 (49.85)	2683 (43.58)	168,017 (49.96)	
Education, *n* (%)				<0.001
High school or below	164,308 (47.98)	3707 (60.22)	160,601 (47.76)	
College degree or above	178,129 (52.02)	2449 (39.78)	175,680 (52.24)	
Smoking status, *n* (%)				<0.001
Never	192,965 (56.35)	2628 (42.69)	190,337 (56.60)	
Previous	115,621 (33.76)	2492 (40.48)	113,129 (33.64)	
Current	33,851 (9.89)	1036 (16.83)	32,815 (9.76)	
Moderate alcohol intake, *n* (%)				<0.001
Yes	172,207 (50.29)	2740 (44.51)	169,467 (50.39)	
No	170,230 (49.71)	3416 (55.49)	166,814 (49.61)	
Physical activity, *n* (%)				<0.001
Low	61,044 (17.83)	1367 (22.21)	59,677 (17.75)	
Moderate	139,634 (40.78)	2393 (38.87)	137,241 (40.81)	
High	141,759 (41.40)	2396 (38.92)	139,363 (41.44)	
Baseline hypertension, *n* (%)				<0.001
Yes	81,425 (23.78)	3134 (50.91)	78,291 (23.28)	
No	261,012 (76.22)	3022 (49.09)	257,990 (76.72)	
Baseline dyslipidemia, *n* (%)				<0.001
Yes	35,734 (10.44)	1601 (26.01)	34,133 (10.15)	
No	306,703 (89.56)	4555 (73.99)	302,148 (89.85)	
KDM-BA, years *	51.92 (12.50)	61.82 (11.82)	51.75 (12.44)	<0.001
PhenoAge, years *	49.12 (9.16)	56.33 (8.32)	49.00 (9.12)	<0.001
KDM-BA acceleration, years *	−3.71 (9.83)	1.06 (11.00)	−3.79 (9.79)	<0.001
PhenoAge acceleration, years *	−6.51 (4.28)	−4.43 (5.09)	−6.54 (4.25)	<0.001

Abbreviation: CMM, cardiometabolic multimorbidity; KDM-BA, Klemera–Doubal method biological age; PhenoAge, phenotypic age. Data are expressed as mean (standard deviation) or numbers (percentage). ^Δ^, High economic level was defined as Townsend Deprivation Index < −2.30 (median value) while low economic level was defined as Townsend Deprivation Index ≥ −2.30. *, The assessment of biological age included 234,184 participants.

**Table 2:** Association of body roundness index with the risk of cardiometabolic multimorbidity (N = 342,437).

Body Roundness Index	Case	Model I		Model II		Model III	
		HR (95% CI)	*p*-Value	HR (95% CI)	*p*-Value	HR (95% CI)	*p*-Value
Q1	443	Reference	-	Reference	-	Reference	-
Q2	940	1.70 (1.52, 1.91)	<0.001	1.66 (1.48, 1.86)	<0.001	1.57 (1.40, 1.76)	<0.001
Q3	1592	2.61 (2.34, 2.90)	<0.001	2.45 (2.20, 2.73)	<0.001	2.16 (1.94, 2.41)	<0.001
Q4	3181	5.28 (4.77, 5.84)	<0.001	4.69 (4.24, 5.20)	<0.001	3.72 (3.35, 4.13)	<0.001

Abbreviations: HR—hazard ratio; CI—confidence interval; Q—quantile. Model I: Adjusted for age (<60, ≥60 years), sex (female, male), and race. Model II: Adjusted for the variables in Model I and education (college degree or above, high school or below), socioeconomic status (low economic level, high economic level), smoking status (never, previous, or current), moderate alcohol intake (yes, no), and physical activity (low, moderate, or high). Model III: Adjusted for the variables in Model II and baseline hypertension (yes, no), and baseline dyslipidemia (yes, no).

## Data Availability

This study was conducted using the UK Biobank (Application Number 88589). For com-prehensive details on accessing UK Biobank data and the data release schedule, please visit https://www.ukbiobank.ac.uk, accessed on 22 October 2024. The R code for statistical analyses may be obtained by contacting the corresponding author.

## Supplementary Materials

The following supporting information can be downloaded at: https://www.mdpi.com/article/10.3390/nu17081397/s1, Figure S1: The relationship of body mass index with cardiometabolic multimorbidity risk after full adjustment (N = 342,437); Figure S2: ROC curves for visceral fat content and traditional obesity indicators in predicting the risk of cardiometabolic multimorbidity (N = 342,437); Figure S3: Correlation between body roundness index and accelerated biological aging (N = 234,184); Table S1: Definition of cardiometabolic disease; Table S2: The biomarkers used to construct biological ages in the UK Biobank; Table S3: Association of body mass index with the risk of cardiometabolic multimorbidity (N = 342,437); Table S4: Association between body roundness index and accelerated biological aging (N = 234,184); Table S5: Association between accelerated biological aging and the risk of cardiometabolic multimorbidity (N = 234,184); Table S6: Mediated effect of accelerated biological aging on the association between body roundness index and the risk of cardiometabolic multimorbidity (N = 234,184); Table S7: Association between body roundness index and cardiometabolic multimorbidity, stratified by potential modifiers (N = 342,437); Table S8: The results of sensitivity analyses of the association between body roundness index and the risk of cardiometabolic multimorbidity (N = 342,437); Table S9: Association between body roundness index and accelerated biological aging by using inverse probability weighted analysis (N = 234,184); Table S10: Association between accelerated biological aging and the risk of cardiometabolic multimorbidity by using inverse probability weighted analysis (N = 234,184).
