# Supplementary material for: The Relationship between Visceral Fat Accumulation and Risk of Cardiometabolic Multimorbidity: The Roles of Accelerated Biological Aging
Source: Nutrients. 2025 Apr 21;17(8):1397. doi: 10.3390/nu17081397 (PMC12030224; doi:10.3390/nu17081397)
Supplement: Supplementary file 1 [file nutrients-17-01397-s001.zip › nutrients-3568361 - supplementary.pdf]

Table S1. Definition of cardiometabolic disease.

Cardiometabolic disease	Self-reported information	ICD-9 information	ICD-10 information
CHD	6150 (1, 2),<br>20002 (1074, 1075),<br>20008	41271 (410-414),<br>41281	41270 (I20-I25, Z951, Z955),<br>41280,<br>131296, 131297, 131298, 131299, 131300, 131301,<br>131302, 131303, 131304, 131305, 131306, 131307
T2DM	2443 (1), 6153 (3), 6177 (3),<br>20002 (1223),<br>20008	41271 (25000, 25010, 25020,<br>25090),<br>41281	41270 (E11),<br>41280,<br>130708, 130709
Stroke	6150 (3),<br>20002 (1081, 1086, 1491, 1583),<br>20008	41271 (3361, 3623, 430, 431,<br>4329, 4330, 4331, 4332, 4333,<br>4338, 4339, 434,436),<br>41281	41270 (I60, I61, I629, I63, I64, I678, I690, I693,<br>G951, H341, H342, S066),<br>41280,<br>131180, 131181, 131360, 131361, 131362, 131363,<br>131364, 131365, 131366, 131367, 131368, 131369,<br>131370, 131371, 131372, 131373, 131374, 131375,<br>131376, 131377, 131378, 131379

Abbreviations: CHD, coronary heart disease; T2DM, type 2 diabetes; ICD-9, international classification of diseaseversion9; ICD-10, international classification of diseaseversion10.

Cardiometabolic multimorbidity refers to the occurrence of at least two of the above-mentioned diseases.

Table S2. The biomarkers used to construct biological ages in the UK Biobank.

Aging-related biomarkers	UK biobank field ID
Forced expiratory volume in 1-second (L)	3063
Systolic blood pressure, automated reading (mm Hg)	4080
White blood cell (leukocyte) count (1000 cells/uL)	30000
Red blood cell (erythrocyte) distribution width (%)	30070
Lymphocyte percentage (%)	30180
Mean sphered cell volume (fL)	30270
Albumin (g/dL)	30600
Alkaline phosphatase (U/L)	30610
Urea (mg/dL)	30670
Cholesterol (mg/dL)	30690
Creatinine (mg/dL)	30700
C-reactive protein (mg/dL)	30710
Glucose (mg/dL)	30740
Glycated haemoglobin (%)	30750

Table S3. Association of body mass index with the risk of cardiometabolic multimorbidity (N = 342,437).

Body mass index	Case	Model I		Model II		Model III	
		HR (95% CI)	*P*-value	HR (95% CI)	*P*-value	HR (95% CI)	*P*-value
Q1	674	Reference	-	Reference	-	Reference	-
Q2	1,068	1.33 (1.20, 1.46)	<0.001	1.32 (1.20, 1.45)	<0.001	1.24 (1.12, 1.36)	<0.001
Q3	1,572	1.84 (1.68, 2.02)	<0.001	1.77 (1.62, 1.94)	<0.001	1.55 (1.42, 1.70)	<0.001
Q4	2,842	3.67 (3.38, 4.00)	<0.001	3.37 (3.09, 3.67)	<0.001	2.67 (2.45, 2.92)	<0.001

Abbreviations: HR, hazard ratio; CI, confidence interval; Q, quantile.

Model I: Adjusted for age (<60, ≥60 years), sex (female, male), and race (white, others).

Model II: Adjusted for the variables in Model I and education (college degree or above, high school or below), socioeconomic status (low economic level, high economic level), smoking status (never, previous, or current), moderate alcohol intake (yes, no), and physical activity (low, moderate, or high).

Model III: Adjusted for the variables in Model II and baseline hypertension (yes, no), and baseline dyslipidemia (yes, no).

Table S4. Association between body roundness index and accelerated biological aging (N = 234,184).

Body roundness index	Model I		Model II		Model III	
	β (95% CI)	*P*-value	β (95% CI)	*P*-value	β (95% CI)	*P*-value
**KDM-BA acceleration**						
Q1	Reference	-	Reference	-	Reference	-
Q2	2.70 (2.60, 2.81)	<0.001	2.61 (2.50, 2.72)	<0.001	2.47 (2.36, 2.57)	<0.001
Q3	5.02 (4.91, 5.13)	<0.001	4.83 (4.72, 4.94)	<0.001	4.52 (4.41, 4.63)	<0.001
Q4	8.28 (8.17, 8.39)	<0.001	7.97 (7.86, 8.08)	<0.001	7.33 (7.21, 7.44)	<0.001
**PhenoAge acceleration**						
Q1	Reference	-	Reference	-	Reference	-
Q2	0.25 (0.20, 0.30)	<0.001	0.21 (0.17, 0.26)	<0.001	0.20 (0.15, 0.25)	<0.001
Q3	0.67 (0.62, 0.72)	<0.001	0.60 (0.55, 0.65)	<0.001	0.57 (0.52, 0.62)	<0.001
Q4	1.71 (1.66, 1.76)	<0.001	1.58 (1.53, 1.63)	<0.001	1.51 (1.46, 1.56)	<0.001

Abbreviations: β, beta coefficient; CI, confidence interval; KDM-BA, Klemera-Doubal method biological age; Q, quantile; PhenoAge, phenotypic age.

Model I: Adjusted for age (<60, ≥60 years), sex (female, male), and race (white, others).

Model II: Adjusted for the variables in Model I and education (college degree or above, high school or below), Townsend Deprivation Index (low economic level, high economic level), smoking status (never, previous, or current), moderate alcohol intake (yes, no), and IPAQ (low, moderate, or high).

Model III: Adjusted for the variables in Model II and baseline hypertension (yes, no), and baseline dyslipidemia (yes, no).

Table S5. Association between accelerated biological aging and the risk of cardiometabolic multimorbidity (N = 234,184).

Accelerated biological aging	Case	Model I		Model II		Model III	
		HR (95% CI)	*P*-value	HR (95% CI)	*P*-value	HR (95% CI)	*P*-value
**KDM-BA acceleration**							
Continuous	3,987	1.05 (1.05, 1.05)	<0.001	1.05 (1.04, 1.05)	<0.001	1.04 (1.04, 1.04)	<0.001
Q1	581	Reference	-	Reference	-	Reference	-
Q2	724	1.52 (1.36, 1.70)	<0.001	1.46 (1.30, 1.62)	<0.001	1.37 (1.23, 1.53)	<0.001
Q3	952	2.14 (1.93, 2.38)	<0.001	1.99 (1.79, 2.20)	<0.001	1.81 (1.63, 2.01)	<0.001
Q4	1,730	4.06 (3.69, 4.46)	<0.001	3.56 (3.23, 3.92)	<0.001	3.07 (2.78, 3.38)	<0.001
**PhenoAge acceleration**							
Continuous	3,987	1.07 (1.07, 1.08)	<0.001	1.07 (1.06, 1.07)	<0.001	1.06 (1.06, 1.07)	<0.001
Q1	539	Reference	-	Reference	-	Reference	-
Q2	734	1.25 (1.12, 1.40)	<0.001	1.23 (1.10, 1.37)	<0.001	1.21 (1.08, 1.35)	0.001
Q3	1,029	1.68 (1.51, 1.87)	<0.001	1.59 (1.44, 1.77)	<0.001	1.56 (1.40, 1.73)	<0.001
Q4	1,685	2.73 (2.47, 3.01)	<0.001	2.39 (2.16, 2.64)	<0.001	2.25 (2.03, 2.48)	<0.001

Abbreviations: HR, hazard ratio; CI, confidence interval; KDM-BA, Klemera-Doubal method biological age; Q, quantile; PhenoAge, phenotypic age.

Model I: Adjusted for age (<60, ≥60 years), sex (female, male), and race (white, others).

Model II: Adjusted for the variables in Model I and education (college degree or above, high school or below), Townsend Deprivation Index (low economic level, high economic level), smoking status (never, previous, or current), moderate alcohol intake (yes, no), and IPAQ (low, moderate, or high).

Model III: Adjusted for the variables in Model II and baseline hypertension (yes, no), and baseline dyslipidemia (yes, no).

Table S6. Mediated effect of accelerated biological aging on the association between body roundness index and the risk of cardiometabolic multimorbidity (N = 234,184).

Mediating factors	Total effect		Indirect effect		Direct effect		Proportion mediated, %
	HR (95% CI)	*P*-value	HR (95% CI)	*P*-value	HR (95% CI)	*P*-value	
**KDM-BA acceleration**							
BRI-Q2	1.52 (1.32, 1.75)	<0.001	1.09 (1.08, 1.10)	<0.001	1.39 (1.21, 1.60)	<0.001	24.26 (17.48, 31.05)
BRI-Q3	2.07 (1.81, 2.36)	<0.001	1.17 (1.16, 1.19)	<0.001	1.76 (1.54, 2.01)	<0.001	28.49 (24.38, 32.61)
BRI-Q4	3.75 (3.31, 4.26)	<0.001	1.29 (1.27, 1.32)	<0.001	2.90 (2.55, 3.30)	<0.001	30.97 (28.39, 33.55)
**PhenoAge acceleration**							
BRI-Q2	1.50 (1.30, 1.72)	<0.001	1.01 (1.01, 1.01)	<0.001	1.48 (1.29, 1.71)	<0.001	3.24 (2.02, 4.47)
BRI-Q3	2.03 (1.78, 2.32)	<0.001	1.03 (1.03, 1.04)	<0.001	1.97 (1.72, 2.25)	<0.001	6.02 (4.94, 7.10)
BRI-Q4	3.70 (3.26, 4.21)	<0.001	1.09 (1.08, 1.09)	<0.001	3.41 (3.00, 3.87)	<0.001	10.83 (9.68, 11.98)

Abbreviations: HR, hazard ratio; CI, confidence interval; KDM-BA, Klemera-Doubal method biological age; PhenoAge, phenotypic age; BRI, body roundness index; Q, quantile.

Using participants within the lowest quartile (Q1) of body roundness index as the reference group.

Confidence intervals were calculated according to the delta method procedure.

All models were adjusted for age (<60, ⩾60 years), sex (female, male), race (white, others), education (college degree or above, high school or below), socioeconomic status (low economic level, high economic level), smoking status (never, previous, or current), moderate alcohol intake (yes, no), physical activity (low, moderate, or high), baseline hypertension (yes, no), and baseline dyslipidemia (yes, no).

Table S7. Association between body roundness index and cardiometabolic multimorbidity, stratified by potential modifiers (N = 342,437).

Subgroup	Simple size	HR (95% CI)	*P*-value
Sex			
Female	185,760	2.38 (2.16, 2.61)	<0.001
Male	156,677	2.00 (1.84, 2.17)	<0.001
Age, years			
<60	208,483	2.82 (2.53, 3.15)	<0.001
≥60	133,954	1.88 (1.74, 2.03)	<0.001
Race			
White	312,134	2.16 (2.02, 2.30)	<0.001
Others	30,303	2.51 (2.03, 3.11)	<0.001
BMI, kg/m^2			
<30	270,502	1.72 (1.60, 1.85)	<0.001
≥30	71,935	2.22 (1.34, 3.70)	<0.001
Economic			
Low economic level	171,737	2.19 (2.01, 2.39)	<0.001
High economic level	170,700	2.18 (1.98, 2.39)	<0.001
Education			
High school or below	164,308	2.14 (1.97, 2.33)	<0.001
College degree or above	178,129	2.25 (2.04, 2.47)	<0.001
Smoking status			
Never	192,965	2.23 (2.03, 2.44)	<0.001
Current or ever	149,472	2.10 (1.92, 2.29)	<0.001
Alcohol consumption			
Moderate intake	172,207	2.05 (1.87, 2.24)	<0.001
Immoderate intake	170,230	2.32 (2.12, 2.53)	<0.001
Physical activity			
Active	281,393	2.18 (2.03, 2.33)	<0.001
Inactive	61,044	2.23 (1.92, 2.60)	<0.001

Abbreviations: HR, hazard ratio; CI, confidence interval; BMI, body mass index.

All models were adjusted for age (<60, ⩾60 years), sex (female, male), race (white, others), education (college degree or above, high school or below), Townsend Deprivation Index (low economic level, high economic level), smoking status (never, previous, or current), moderate alcohol intake (yes, no), physical activity (low, moderate, or high), baseline hypertension (yes, no), and baseline dyslipidemia (yes, no).

Table S8. The results of sensitivity analyses of the association between body roundness index and the risk of cardiometabolic multimorbidity (N = 342,437).

Q1		Q2		Q3		Q4	
**HR (95% CI)**	***P* value**	**HR (95% CI)**	***P* value**	**HR (95% CI)**	***P* value**	**HR (95% CI)**	***P* value**
**Sensitivity analysis 1: A competing risks model was employed.**							
Reference	-	1.58 (1.41, 1.78)	<0.001	2.19 (1.96, 2.44)	<0.001	3.72 (3.35, 4.13)	<0.001
**Sensitivity analysis 2: Excluding participants with CMM diagnosed within two years since enrollment.**							
Reference	-	1.58 (1.40, 1.77)	<0.001	2.17 (1.94, 2.42)	<0.001	3.71 (3.34, 4.13)	<0.001
**Sensitivity analysis 3: Excluding participants with cancer at baseline.**							
Reference	-	1.67 (1.48, 1.89)	<0.001	2.28 (2.02, 2.56)	<0.001	3.97 (3.54, 4.44)	<0.001
**Sensitivity analysis 4: Excluding participants without complete covariates data.**							
Reference	-	1.56 (1.39, 1.75)	<0.001	2.15 (1.93, 2.40)	<0.001	3.69 (3.33, 4.10)	<0.001

Abbreviations: Q, quantile; HR, hazard ratio; CI, confidence interval.

All models were adjusted for age (<60, ⩾60 years), sex (female, male), race (white, others), education (college degree or above, high school or below), Townsend Deprivation Index (low economic level, high economic level), smoking status (never, previous, or current), moderate alcohol intake (yes, no), IPAQ (low, moderate, or high), baseline hypertension (yes, no), and baseline dyslipidemia (yes, no).

Table S9. Association between body roundness index and accelerated biological aging by using inverse probability weighted analysis (N = 234,184).

Body roundness index	β (95% CI)	*P*-value
**KDM-BA acceleration**		
Q1	Reference	-
Q2	2.46 (2.36, 2.57)	<0.001
Q3	4.51 (4.40, 4.62)	<0.001
Q4	7.30 (7.19, 7.42)	<0.001
**PhenoAge acceleration**		
Q1	Reference	-
Q2	0.20 (0.15, 0.25)	<0.001
Q3	0.57 (0.52, 0.62)	<0.001
Q4	1.52 (1.47, 1.57)	<0.001

Abbreviations: β, beta coefficient; CI, confidence interval; KDM-BA, Klemera-Doubal method biological age; Q, quantile; PhenoAge, phenotypic age.

All models were adjusted for age (<60, ⩾60 years), sex (female, male), race (white, others), education (college degree or above, high school or below), Townsend Deprivation Index (low economic level, high economic level), smoking status (never, previous, or current), moderate alcohol intake (yes, no), IPAQ (low, moderate, or high), baseline hypertension (yes, no), and baseline dyslipidemia (yes, no).

Table S10. Association between accelerated biological aging and the risk of cardiometabolic multimorbidity by using inverse probability weighted analysis (N = 234,184).

Accelerated biological aging	HR (95% CI)	*P*-value
**KDM-BA acceleration**		
Continuous	1.04 (1.04, 1.04)	<0.001
Q1	Reference	-
Q2	1.37 (1.22, 1.53)	<0.001
Q3	1.80 (1.62, 2.01)	<0.001
Q4	3.06 (2.77, 3.37)	<0.001
**PhenoAge acceleration**		
Continuous	1.06 (1.06, 1.07)	<0.001
Q1	Reference	-
Q2	1.21 (1.08, 1.35)	0.001
Q3	1.56 (1.40, 1.73)	<0.001
Q4	2.25 (2.04, 2.49)	<0.001

Abbreviations: HR, hazard ratio; CI, confidence interval; KDM-BA, Klemera-Doubal method biological age; Q, quantile; PhenoAge, phenotypic age.

All models were adjusted for age (<60, ⩾60 years), sex (female, male), race (white, others), education (college degree or above, high school or below), Townsend Deprivation Index (low economic level, high economic level), smoking status (never, previous, or current), moderate alcohol intake (yes, no), IPAQ (low, moderate, or high), baseline hypertension (yes, no), and baseline dyslipidemia (yes, no).

Figure S1. The relationship of body mass index with cardiometabolic multimorbidity risk after full adjustment (N = 342,437).

The solid curve line represents the effect-size estimates for the association, and the light shadow represents the 95% confidence interval.

All models were adjusted for age (<60, ≥60 years), sex (female, male), race (white, others), education (college degree or above, high school or below), Townsend Deprivation Index (low economic level, high economic level), smoking status (never, previous, or current), moderate alcohol consumption (yes, no), IPAQ (low, moderate, or high), baseline hypertension (no, yes), and baseline dyslipidemia (no, yes).

Figure S2. ROC curves for visceral fat content and traditional obesity indicators in predicting the risk of cardiometabolic multimorbidity (N = 342,437).

Abbreviations: ROC, receiver operating characteristic curve; AUC, area under the curve; 95%CI, 95% confidence interval; BRI, body roundness index; BMI, body mass index.

Figure S3. Correlation between body roundness index and accelerated biological aging (N = 234,184).

Abbreviations: KDM-BA, Klemera-Doubal method biological age; PhenoAge, phenotypic age.
