# Supplementary figures and images for: The Relationship between Visceral Fat Accumulation and Risk of Cardiometabolic Multimorbidity: The Roles of Accelerated Biological Aging
Source: Nutrients. 2025 Apr 21;17(8):1397. doi: 10.3390/nu17081397 (PMC12030224; doi:10.3390/nu17081397)

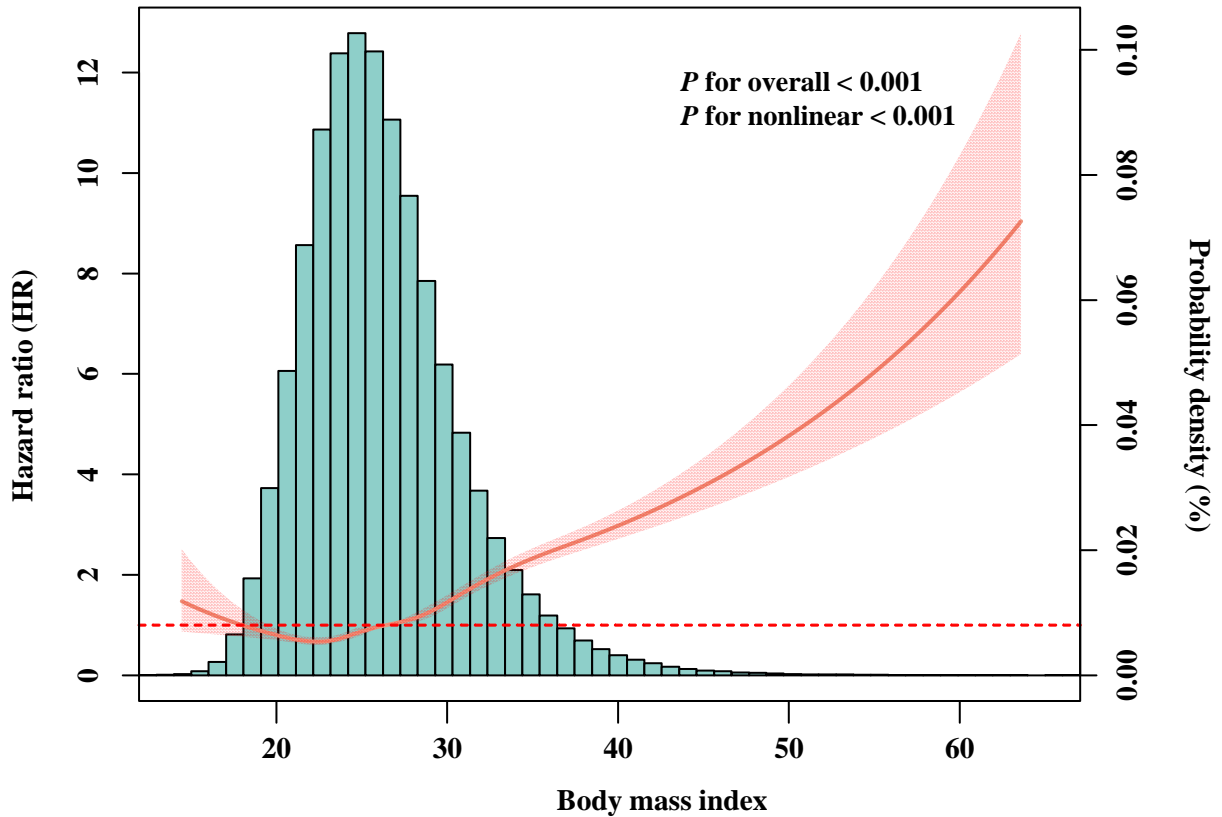

Supplement: Supplementary file 1 [file nutrients-17-01397-s001.zip › FigureS1.pdf]

Comparison of two ROC curves

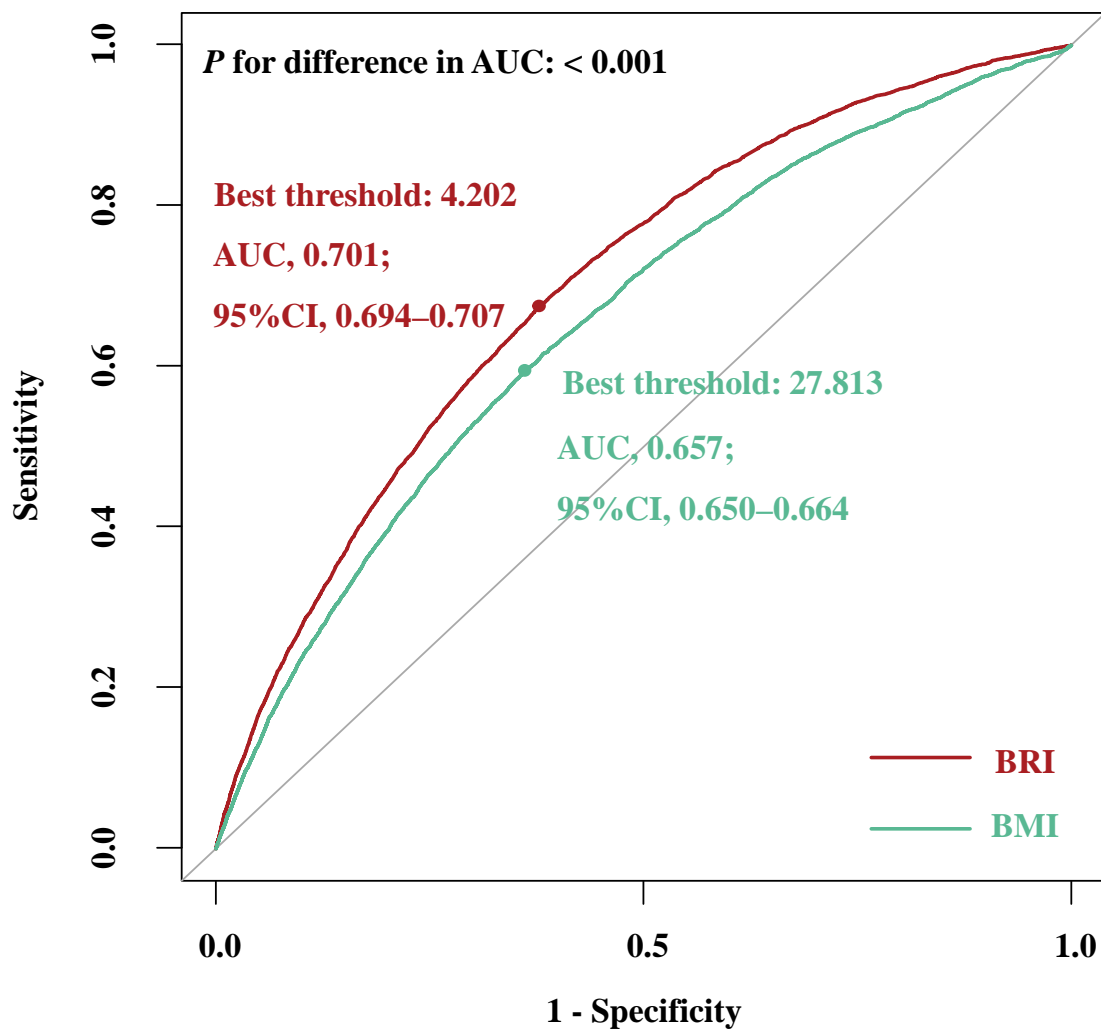

Supplement: Supplementary file 1 [file nutrients-17-01397-s001.zip › FigureS2.pdf]

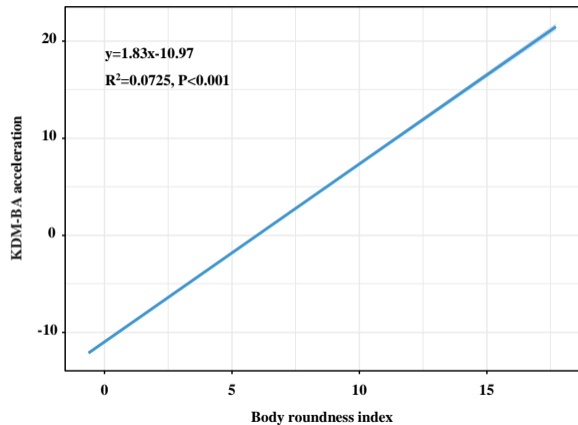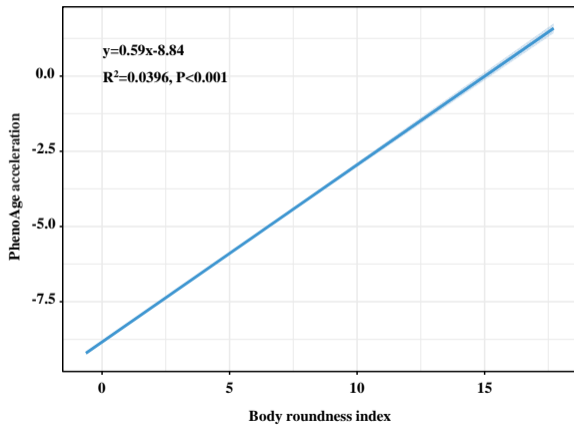

Supplement: Supplementary file 1 [file nutrients-17-01397-s001.zip › FigureS3.pdf]
